# A Cross-Sectional Study of the Association between Infant Hepatitis B Vaccine Exposure in Boys and the Risk of Adverse Effects as Measured by Receipt of Special Education Services

**DOI:** 10.3390/ijerph15010123

**Published:** 2018-01-12

**Authors:** David A. Geier, Janet K. Kern, Kristin G. Homme, Mark R. Geier

**Affiliations:** 1 Institute of Chronic Illnesses, Inc., Silver Spring, MD 20905, USA; davidallengeier@comcast.net (D.A.G.); mgeier@comcast.net (M.R.G.); 2 CoMeD, Inc., Silver Spring, MD 20905, USA; 3 Council for Nutritional and Environmental Medicine (CONEM), US Autism Research Group, Allen, TX 75013, USA; 4 International Academy of Oral Medicine and Toxicology, Champions Gate, FL 33896, USA; khomme@sbcglobal.net

**Keywords:** autism, special education services, developmental delay, ethylmercury, learning disabilities, Merthiolate, Thiomersal

## Abstract

The National Center for Education Statistics reported that between 1990–2005 the number of children receiving special education services (SES) rose significantly, and then, from 2004–2012, the number declined significantly. This coincided with the introduction of Thimerosal-containing hepatitis B vaccine in 1991, and the subsequent introduction of Thimerosal-reduced hepatitis B vaccine in the early 2000s. This study examined the potential relationship between infant exposure to mercury from three doses of Thimerosal-containing hepatitis B vaccine and the risk of boys being adversely affected (as measured by receipt of SES). This cross-sectional study examined 1192 boys (weighted *n* = 24,537,123) 7–8 years of age (born: 1994–2007) from the combined 2001–2014 National Health and Nutritional Examination Survey (NHANES). Survey logistic regression modeling revealed that an exposed population receiving three doses of infant Thimerosal-containing hepatitis B vaccine (weighted *n* = 11,186,579), in comparison to an unexposed population (weighted *n* = 704,254), were at an increased risk of receipt of SES. This association was robust (crude odds ratio = 10.143, *p* = 0.0232), even when considering covariates, such as race and socioeconomic status (adjusted odds ratio = 9.234, *p* = 0.0259). Survey frequency modeling revealed that receipt of SES for the population that was exposed to three doses of Thimerosal-containing hepatitis B vaccine in infancy (12.91%) was significantly higher than the unexposed population (1.44%) (prevalence ratio = 8.96, *p* = 0.006, prevalence attributable rate = 0.1147). Despite the limitation of this cross-sectional study not being able to ascribe a direct cause-and-effect relationship between exposure and outcome, it is estimated that an additional 1.2 million boys received SES with excess education costs of about United States (US) $180 billion associated with exposure to Thimerosal-containing hepatitis B vaccine. By contrast, exposure to Thimerosal-reduced hepatitis B vaccine was not associated with an increased risk of receiving SES. Therefore, routine childhood vaccination is important to reduce the morbidity and mortality of infectious diseases, but every effort should be made to eliminate Thimerosal from all vaccines.

## 1. Introduction

In 1975, the Individuals with Disabilities Education Act (IDEA) mandated that a child who has a disability adversely impacting academic performance and who is in need of special education and related services should receive a free and appropriate public school education. Special education services include different disability categories or types. Most special education services cover a specific learning disability and/or speech/language impairment. The bulk of the remainder covers other health impairments, including autism, intellectual disability, developmental delay, and emotional disturbance. The remaining small percentage includes multiple disabilities, hearing impairment, and orthopedic impairment. Data collection activities under IDEA began in 1976 and as such, data on the numbers of children and youth receiving special education services over the years is publicly available.

According to the National Center for Education Statistics, “From school years 1990–1991 through 2004–2005, the number of children and youth ages 3–21 who received special education services increased from 4.7 million, or 11 percent of total public school enrollment, to 6.7 million, or 14 percent of total public school enrollment. Both the number and percentage of children and youth served under IDEA declined from 2004–2005 through 2011–2012. The number and percentage of children and youth served appeared to level off between 2012–2013 and 2014–2015. By 2014–2015, the number of children and youth served under IDEA was 6.6 million, or 13 percent of total public school enrollment” [1].

The increase in the number of children and youth ages 3–21 who received special education services between school years 1990–1991 through 2004–2005 appears to coincide with the recommendation issued by the United States Centers for Disease Control and Prevention (US CDC) to administer three doses of Thimerosal-containing hepatitis B vaccine routinely to American infants beginning in 1991 [2].

Thimerosal is an organic mercury (Hg)-containing compound (49.55% Hg by weight) that was used as a preservative in certain vaccines, including hepatitis B in the 1990s. Thimerosal rapidly dissociates in aqueous solutions to release ethyl-Hg hydroxide, ethyl-Hg chloride, and thiosalicylate [3]. Infants in the 1990s received 12.5 micrograms (μg) Hg per dose of Thimerosal-containing hepatitis B vaccine, and consequently received a total of 37.5 μg Hg from the three dose series. The dosing schedule was recommended to begin at birth and to be completed by six months of age [2]. Thus, in the 1990s, the infant hepatitis B vaccine was often the earliest routine vaccine source of Thimerosal exposure.

Then in July of 1999, the American Academy of Pediatrics (AAP) and the United States Public Health Service (USPHS) issued a joint recommendation to remove Thimerosal from all childhood vaccines as soon as possible [4]. Vaccine manufacturers began to license, manufacture, and distribute Thimerosal-reduced (<1 μg Hg per dose) childhood vaccines in the US. The decline in the percentage of students served under the IDEA from 2004–2005 through 2011–2012 appears to coincide with the reduction of Thimerosal from some childhood vaccines, including hepatitis B that were routinely administered to American infants beginning in 2001.

Given this context, the present study examined the National Health and Nutrition Examination Survey (NHANES) data to undertake a cross-sectional study to evaluate the potential relationship between infant exposure to Hg from Thimerosal-containing hepatitis B vaccines and the long-term risk of boys being adversely affected, as measured by receipt of special education services. These analyses were undertaken because the only source of potential infant exposure to Hg from Thimerosal-containing childhood vaccines consistently measured across all of the years of NHANES data examined was hepatitis B vaccine. In addition, boys were exclusively examined because previous studies have revealed males were more susceptible to the toxic effects of Hg than females [5]. It was hypothesized that infant exposure to Hg from three doses of Thimerosal-containing hepatitis B vaccines, in comparison to no exposure to hepatitis B vaccines, among children born in the 1990s, would significantly increase the long-term risk to boys of adverse effects, as measured by receipt of special education services. It was also hypothesized that the subsequent reduction of Thimerosal from hepatitis B vaccines in the early 2000s would reduce the long-term risk of boys incurring adverse effects as measured by receipt of special education services following infant exposure to three doses of hepatitis B vaccine, in comparison to no doses of hepatitis B vaccine, among children born in the 2000s.

## 2. Methods

This study employed Statistical Analysis Software (SAS; Version 9.4; SAS Institute Inc., 100 SAS Campus Drive, Cary, NC, USA) running on a 64-bit based PC with dual core Intel^®^ (Santa Clara, CA, USA) Xeon^®^ CPU x5680 at 3.33 GHz, 6 cores, and 12 logical processors, with 44.0 GB of RAM, and utilizing Microsoft (Redmond, WA, USA) Windows 7 Ultimate operating system to examine the NHANES data. This study integrated NHANES data including demographic, socioeconomic, immunization, and health-related questions.

### 2.1. Study Participants

An overall population of 1192 boys born from 1994–2007 between the ages of 7 and 8 years old, with non-missing values for the demographic, immunization, and special education status variables that were examined in this study was created by combining data from the 2001–2014 NHANES. These years were selected because they were derived from the most recently available NHANES data for analyses. Then, to this population, the full sample two-year interview weight variable (variable: WTINT2YR) was applied, yielding a population of 24,537,123 American boys represented in this study.

In NHANES, a sample weight is assigned to each sample participant. The sample weight is a measure of the number of persons in the target population that the sampled individual represents. Sample weights are needed to obtain unbiased estimates of population parameters when the sample participants are chosen with unequal probabilities.

The demographic variables for the population were identified from within the NHANES demographic dataset. The variables examined were as follows: gender (variable: RIAGENDR, male = 1), age in years at screening (variable: RIDAGEYR, values = 7–8), race (variable: RIDRETH2, non-Hispanic White = 1, non-Hispanic Black = 2, and Hispanic = 3 (Mexican American + Other Hispanic)), and socioeconomic status (variable: INDFMPIR, values = 0–5, poverty income ratio (PIR)—a ratio of family income to poverty threshold).

### 2.2. Outcomes

The physical functioning NHANES dataset was examined for the special education status of each person in the population. Each person had an identified status for special education services (variable: PFQ40 or PFQ41 [Yes = 1, No = 2], survey question, “Does {study participant} receive Special Education or Early Intervention Services”?).

### 2.3. Exposures

The immunization exposure variable was identified from within the NHANES immunization dataset. The variable examined was hepatitis B vaccine (variable: IMQ020, survey question, “Have {study participant} ever received the 3-dose series of the hepatitis B vaccine”?). Persons identified as receiving three doses of hepatitis B vaccine (variable: IMQ020 = 1) and persons identified as receiving no doses of hepatitis B vaccine (variable: IMQ020 = 3) were included in the population examined in this study. Persons identified as receiving two doses of hepatitis B vaccine were excluded (this is a very small minority of persons). 

The population was divided into three different contemporaneous population groups. The first group was the overall population composed of all persons born from 1994–2007 (*n* = 1192, weighted *n* = 24,537,123). The overall infant hepatitis B vaccine exposed population was defined as those persons receiving three doses of hepatitis B vaccine (variable: hepatitis B vaccine exposure = 2, *n* = 1115, weighted *n* = 22,994,525) and the overall unexposed population was defined as those persons receiving no doses of hepatitis B vaccine (variable: hepatitis B vaccine exposure = 1, *n* = 77, weighted *n* = 1,542,598).

The overall population was then divided into two sub-populations based on the presumed Thimerosal status of their infant hepatitis B vaccines. Historically, Thimerosal-containing infant hepatitis B vaccines were routinely recommended for administration to infants beginning in 1991 [2], through approximately the year 2000 [6]. The second group examined in this study was the sub-population of persons born from 1994–2000 (*n* = 565, weighted *n* = 11,890,832). Among this sub-population, the infant Thimerosal-containing hepatitis B vaccine exposed group was defined as those persons receiving three doses of hepatitis B vaccine (variable: hepatitis B vaccine exposure = 2, *n* = 524, weighted *n* = 11,186,579), and the unexposed group was defined as those persons receiving no doses of hepatitis B vaccine (variable: hepatitis B vaccine exposure = 1, *n* = 41, weighted *n* = 704,254). The third group examined in this study was the sub-population of persons born from 2001–2007 (*n* = 627, weighted *n* = 12,646,291). Among this sub-population, the infant Thimerosal-reduced hepatitis B vaccine exposed group was defined as those persons receiving three doses of hepatitis B vaccine (variable: hepatitis B vaccine exposure = 2, *n* = 591, weighted *n* = 11,807,946) and the unexposed group was defined as those persons receiving no doses of hepatitis B vaccine (variable: hepatitis B vaccine exposure = 1, *n* = 36, weighted *n* = 838,345).

### 2.4. Statistical Analyses

In all statistical analyses, the statistical package in SAS was utilized, and a two-sided *p*-value < 0.05 was considered statistically significant. The null hypothesis was that there would be no relationship between infant hepatitis B vaccine exposure and the risk of adverse effects, as measured by receipt of special education services.

Initially, using the survey logistic regression modeling procedure in SAS (stratum-variable: SDMVSTRA, cluster-variable: SDMVPSU, and weight-variable: WTINT2YR), the potential relationship between infant hepatitis B vaccine exposure and the risk for adverse effects as measured by receipt of special education services was examined for the three different exposure groups (Model I). Then, a survey logistic regression model procedure in SAS was developed to include, in addition to hepatitis B vaccine exposure, other standard covariates such as race and socioeconomic status, to determine what, if any, effect these covariates had on the model for the three different exposure groups examined (Model II). Finally, the survey frequency modeling procedure in SAS (stratum-variable: SDMVSTRA, cluster-variable: SDMVPSU, and weight-variable: WTINT2YR) was employed to evaluate the potential relationship between hepatitis B vaccine exposure and the risk for adverse effects as measured by receipt of special education services for sub-population groups two and three (Model III). The Rao-Scott χ^2^ test statistic was employed to determine statistical significance. The prevalence ratio = (weighted number of boys receiving special education services in the exposed group/weighted total number of boys in the exposed group)/(weighted number of boys receiving special education services in the unexposed group/weighted total number of boys in the unexposed group). The prevalence attributable rate = (weighted total number of boys receiving special education services in the exposed group/weighted total number of boys in the exposed group) − (weighted total number of boys receiving special education services in the unexposed group/weighted total number of boys in the unexposed group). The weighted total number of boys receiving special education services attributably associated to exposure = weighted total number of boys in the exposed group × the prevalence attributable rate.

## 3. Results

Table 1 displays the characteristics of the overall population of boys born from 1994–2007 that were examined in this study (*n* = 1192, weighted *n* = 24,537,123). The overall exposed group receiving three doses of infant hepatitis B vaccine (*n* = 1115, weighted *n* = 22,994,525) was similar to the overall unexposed group receiving no doses of hepatitis B vaccine (*n* = 77, weighted *n* = 1,542,598) for age, birth year, race, and socioeconomic status.

Table 2 summarizes the survey logistic regression models generated to examine the potential relationship between the three doses of infant hepatitis B vaccine exposure and the risk of adverse effects, as measured by receipt of special education services. It was revealed that the exposed group receiving three doses of infant Thimerosal-containing hepatitis B vaccine in comparison to its contemporaneous unexposed group receiving no doses of hepatitis B vaccine was at a more than nine-fold significantly higher risk of adverse effects, as measured by receipt of special education services in both the unadjusted model (Model I, odds ratio (OR) = 10.143, *p* = 0.0232, 95% confidence interval (CI) = 1.373 to 74.950) and in the adjusted model (Model II, OR = 9.234, *p* = 0.0259, 95% CI = 1.306 to 65.286). In addition, no differences in the risk for adverse effects, as measured by receipt of special education services among boys were observed when comparing the overall exposed group that received three doses of infant hepatitis B vaccine (regardless of Thimerosal status) in the unadjusted model (Model I, OR = 1.707, *p* = 0.2551, 95% CI = 0.680 to 4.286), or the adjusted model (Model II, OR = 1.686, *p* = 0.2722, 95% CI = 0.664 to 4.285), or the exposed group that received three doses of infant Thimerosal-reduced hepatitis vaccine in the unadjusted model (Model I, OR = 0.977, *p* = 0.9647, 95% CI = 0.348 to 2.744) or the adjusted model (Model II, OR = 0.952, *p* = 0.9286, 95% CI = 0.324 to 2.798) as compared to their respective contemporaneous unexposed groups receiving no doses of hepatitis B vaccine. Finally, it was revealed in Model II that receipt of special education services was also significantly related to the covariates of race (non-Hispanic Whites were significantly more likely to receive special education services than Hispanics) and socioeconomic status in two of the three models constructed.

Table 3 displays the prevalence ratio for receipt of special education services subsequent to the exposed group receiving three doses of infant hepatitis B vaccine in comparison to its contemporaneous unexposed group receiving no doses of hepatitis B vaccine. It was observed that exposure to three doses of infant Thimerosal-containing hepatitis B vaccine, in comparison to no-exposure, significantly increased the risk (prevalence ratio = 8.961, *p* = 0.006, 95% CI = 1.202 to 66.667) and the attributable risk (prevalence attributable rate = 0.1147) of receipt of special education services. As a consequence, it is estimated that 1,283,101 boys born from 1994–2000 received special education services associated with receipt of three doses of infant Thimerosal-containing hepatitis B vaccine. By contrast, it was observed that exposure to three doses of infant Thimerosal-reduced hepatitis B vaccine, in comparison to no-exposure, was not associated with an increased risk or attributable risk of receipt of special education services.

## 4. Discussion

The results observed in this cross-sectional study provide important new epidemiological evidence supporting the hypothesis that was being tested in this study—that infant Hg exposure from three doses of Thimerosal-containing hepatitis B vaccines significantly increased the long-term risk of boys receiving special education services. Notably, this risk was observed among boys born in the 1990s. By contrast, and as hypothesized, it was observed that infant exposure to three doses of Thimerosal-reduced hepatitis B vaccine, which was available in the 2000s, in comparison to no exposure to hepatitis B vaccine, revealed no increased long-term risk of boys receiving special education services. This study confirmed this relationship via statistical models using survey logistic regression, even when including the covariates of race and socioeconomic status.

Many previous epidemiological studies examining Hg exposure from infant Thimerosal-containing vaccines have observed adverse effects similar to those that are observed in the present study. The previous study that most closely resembles the present study is a 2008 study examining the potential relationship between infant Thimerosal-containing hepatitis B vaccine exposure and the long-term risk of receipt of special education services [7]. In the Gallagher and Goodman study, as in the present study, the investigators examined the NHANES data for the rate of special education services among boys receiving three doses of infant Thimerosal-containing hepatitis B vaccines in comparison to no hepatitis B vaccines. They observed, consistent with the significantly increased risk observed in the present study, that boys who received three doses of infant Thimerosal-containing hepatitis B vaccines in comparison to no hepatitis B vaccines incurred a nine-fold significantly increased risk for receiving special education services, after adjustment for confounders. Even though both studies observed a significant relationship between infant exposure to Thimerosal-containing hepatitis B vaccines and the long-term risk for boys of receiving special education services, the present study is differentiated from the previous study by the fact that the investigators in the previous study only examined a single year, 1999–2000, from the NHANES data on children born in the 1990s. As a result, the previous study was not able to test and evaluate the potential consequences of the reduction of Thimerosal from hepatitis B vaccines in the 2000s. In addition, unlike the present study, in which, by combining multiple NHANES years, it was possible to ensure that enough data was collected on older children (i.e., 7–8 year-olds) with greater stability in their rates of special education services, the previous study had to rely on the rates of special education services across a much wider age group (i.e., 1–9 year-olds). Thus, the wider age group examined in the previous study may have impacted the stability of the rate of special education services examined (i.e., different age groups may receive different rates of special education services). Finally, the present study is differentiated from the previous study because the present study provides an estimate of the total number of boys in the US who received special education services that are attributably associated with the administration of three doses of infant Thimerosal-containing hepatitis B vaccines.

A number of other previous epidemiological studies have examined exposures and outcomes similar to those in the present study and have reported results that are compatible with those of the present study. Three of these studies used different statistical methods applied to the Vaccine Safety Datalink (VSD) to evaluate infant Thimerosal-containing hepatitis B vaccine exposure and its relation to outcomes that are common diagnoses among those receiving special education services, such as learning disabilities, autism, and emotional disturbances. For example, a 2016 longitudinal cohort study evaluated the relationship between exposure to Hg from Thimerosal-containing hepatitis B vaccines administered at specific intervals within the first six months of life and the long-term risk of being medically diagnosed with a learning disability [8]. These investigators observed that infants receiving additional doses of Hg from Thimerosal-containing hepatitis B vaccines administered within the first month, the first two months, and the first six months of life, in comparison to infants receiving no Hg doses from Thimerosal-containing hepatitis B vaccines, were at a significantly increased long-term risk of being diagnosed with learning disabilities. These investigators then concluded that from 1991–2001 an estimated 500,000–1 million additional US children were diagnosed with learning disabilities as a consequence of increased exposure to Hg from Thimerosal-containing hepatitis B vaccines, with an additional lifetime cost exceeding US $1 trillion. 

Using a different statistical method, investigators undertook a longitudinal case-control study in the VSD to evaluate the relationship between infant Thimerosal-containing hepatitis B vaccination at specific intervals within the first six months of life and the long-term risk of children being diagnosed with learning disabilities [9]. Like the 2016 study, the 2014 study revealed that increasing doses of Hg from Thimerosal-containing hepatitis B vaccines within the first month, the first two months, and the first six months of life significantly increased the long-term risk of children being diagnosed with learning disabilities. 

Yet another longitudinal case-control study in the VSD undertook to evaluate the potential dose-response relationship between increasing exposure to Hg from infant Thimerosal-containing hepatitis B vaccines administered within the first six months of life and the increasing long-term risk of a child being diagnosed with neurodevelopmental disorders, specifically, learning disabilities, autism, attention-deficit/hyperactivity disorder (ADHD), or tic disorder [10]. It was revealed on a per μg Hg basis, that cases of autism, ADHD, learning disabilities, or tic disorder were significantly more likely than controls to have received increased Hg exposure from Thimerosal-containing hepatitis B vaccines administered within the first six months of life.

It is important to note that the three aforementioned studies examining the relationship between Hg exposure from Thimerosal-containing hepatitis B vaccines and adverse outcomes utilized the VSD. The VSD database is a cornerstone of US post-licensure vaccine safety monitoring, established as a joint collaboration between the CDC and nine integrated healthcare systems [11]. Medical event information, specific vaccine history, and selected demographic information from multiple Health Maintenance Organizations (HMOs) are linked together in the VSD database [12], and it was previously demonstrated that the VSD population is large enough to be representative of the general US population [11].

In addition, other epidemiological studies using the Vaccine Adverse Events Reporting System (VAERS), support the observation in this study that the presence or the reduction of Thimerosal in vaccines is a mediating component for the risk of boys receiving special education services. For example, a 2006 ecological study examined time-trends in neurodevelopmental disorder outcomes reported to the VAERS for vaccines administered from 1991–2004 [6]. It was observed that neurodevelopmental disorders reported to VAERS rose significantly from 1991–1998 (a time when there was a significant increase in Hg exposure due to new recommendations for routine administration of Thimerosal-containing vaccines to infants), and it was also observed that the neurodevelopmental disorders reported to VAERS dropped significantly from mid-1999 onward (a time when there was a significant reduction in Hg exposure due to the adoption of Thimerosal-reduced vaccines). A 2014 case-control study of the VAERS revealed that cases diagnosed with the outcomes of autism, speech disorders, or developmental delay were significantly more likely than controls to have received exposure to a Thimerosal-preserved diphtheria-tetanus-acellular-pertussis (DTaP) vaccine (administered from 1997–1999) in comparison to its Thimerosal-reduced formulation (administered from 2004–2006) [13]. Finally, a 2016 two-phase case-control study revealed a significant reduction in the risk of receiving an autism diagnosis among persons that were born from 1998–2003 [14].

Despite the limitation of this cross-sectional study not being able to ascribe a direct cause-and-effect relationship between exposure and outcome, it was estimated that an additional 1,283,101 boys born from 1994–2000 received special education services associated with receipt of three doses of infant Thimerosal-containing hepatitis B vaccine. The National Education Association has estimated that special education students cost on average an additional $9369 per student per school year [15]. Special education students are entitled to receive special education services from their local school district from age 3–18 or 21 years-old depending upon the locality. Assuming an average student receives 15 years of special education services, it is estimated that the excess overall education cost for the average special education student would be about US $140,535. This would mean that the excess cost of special education services provided to American boys born from 1994–2000 would be US $180,320,599,035 as a consequence of infant exposure to three doses of Thimerosal-containing hepatitis B vaccines.

It was observed from NHANES there was a total of 1,454,735 boys who received special education services that were born from 1994–2000. As revealed from the data in Table 3, it is reasonable to infer that out of the 1,454,735 boys who received special education services and were born from 1994–2000, a total of 1,283,101 boys, or more than 88% (or about 1 in 1.13 boys receiving special education), were associated with exposure to three doses of infant Thimerosal-containing hepatitis B vaccine. This means that the majority of the boys who were receiving special education services and who were born from 1994–2000 were associated with infant Thimerosal-containing hepatitis B vaccine exposures, and were not associated with other environmental and/or genetic factors working in isolation. These results are compatible with several previous studies [8,9,10].

In contrast to the results observed in this study, two previous epidemiological studies failed to find a relationship between increasing Thimerosal-containing vaccine exposure and learning disabilities [16,17]. In the first study, the investigators used a cohort study design to evaluate the relationship between neurodevelopmental disorders, such as learning disabilities and Thimerosal-containing vaccine administration in the VSD database [16]. These investigators found no consistent significant increased risk for learning disabilities following increasing exposures to Thimerosal-containing vaccines. In the second study, the investigators used a cohort study design to evaluate the relationship between neurodevelopmental disorder outcomes and Thimerosal-containing vaccine administration in the general practitioner research database (GPRD) [17]. These investigators observed the non-biologically plausible result that increased exposure to Thimerosal-containing vaccines, in a dose-dependent fashion, significantly reduced the risk of learning disabilities. As described elsewhere, these previously mentioned studies were analyzed in detail for their methodological limitations [18,19].

The results of this study are also biologically plausible. It was previously reported that Thimerosal-containing childhood vaccines were a significant source of Hg exposure for many infants and resulted in some infants having Hg exposures and blood Hg levels that exceeded the safety limits established by several governmental agencies for methyl-Hg, a chemically similar compound [20,21,22,23]. No regulatory safety standard has been set for ethyl-Hg, although ethyl-Hg’s mechanisms of toxicity are considered to be similar to that for methyl-Hg, according to a recent study by the US CDC [24]. Furthermore, it has been previously reported that alkyl-Hg compounds such as ethyl-Hg are known human developmental toxicants when exposure occurs in the prenatal and early postnatal periods [5]. Further, consistent with the adverse effects observed in this study from infant Thimerosal-containing hepatitis B vaccines and the risk of receiving special education services in boys, it was reported that susceptibility to Hg toxicity is greater in younger persons and in males relative to females [5]. 

Moreover, one of the detrimental effects of Hg exposure in the brain is axonal degeneration or axon loss. Of particular concern is the loss of long-range axons because, in general, once lost, they do not regenerate; i.e., they do not restore their original connection [25]. Long-range connections are important in many processes in the brain, such as reading, hearing, coordination, and speech/language [26,27,28,29,30,31], and abnormalities in long-range neural tracts are associated with delays in development. Literacy, speech, and learning require these more speed-efficient long-range neural networks to bring about efficient communication overall between these regions [26,27,28,29,30,31]. 

## 5. Strengths/Limitations

An important strength of this study was its examination of children from the NHANES program. The NHANES is a program of studies designed to assess the health and nutritional status of adults and children in the US. The survey is unique in that it combines interviews with physical and laboratory examinations. NHANES is a major program of the National Center for Health Statistics (NCHS) of the US CDC. The NHANES program began in the early 1960s and has been conducted as a series of surveys focusing on different population groups or health topics. In 1999, the survey became a continuous program that has adaptable components on a variety of health and nutritional measurements to meet emerging needs. The NHANES examines a nationally representative sample of about 5000 Americans each year, and these persons are located in counties across the US, 15 of which are visited each year. As described by NCHS, and consistent with the objective of this study, NHANES is supposed to be used in epidemiological studies to determine the prevalence of major diseases and risk factors for diseases, which can help to develop sound public health policy.

A further strength of this study is that an examination of the NHANES data on boys receiving special education services revealed an overall rate of 13.01%, which is comparable to the established US population rate of special education services reported from the IDEA data. As described previously, the IDEA data revealed that the percentage of students receiving special education services ranged from 11–14% for school years 1990–1991 through 2004–2005 [1].

Another important strength of this study was the special emphasis placed on the age of the population examined—boys aged 7–8 years. These ages were chosen so as to ensure that the boys were sufficiently old enough to have received diagnoses and to have entered into school-based special education service programs, but not so old as to have aged-out of these programs. This age range is consistent with that used by the US CDC to examine populations for the presence of neurodevelopmental outcomes [4].

An additional strength of this study was that the NHANES data was collected independently of the methods used. Further, the different NHANES data elements were also collected independently from one another (i.e., persons providing information about their special education services status did not know that this would be related to information collected about hepatitis B immunization status). As a result, biases such as selection bias for study participants or recall bias or examiner bias should have minimally impacted the results observed in this study.

A further strength of this study was the consistency and specificity of the results observed. It was consistently observed in this study that exposure to three doses of infant Thimerosal-containing hepatitis B vaccine were associated with an increased risk of boys receiving special education services. This association remained consistent when using different statistical methods, and when including complex covariates. The specificity of the results observed in this study was further confirmed when the statistical models revealed no significant relationship between exposure to three doses of infant Thimerosal-reduced hepatitis B vaccine and boys receiving special education services.

A potential limitation of this study was that, as with any cross-sectional study, it was not possible to ascribe a direct cause-and-effect relationship between exposure and outcome. Despite this limitation, the results that are observed in this study, as described previously, are biologically plausible and are supported by a number of aforementioned epidemiological studies linking increased Hg exposure with boys receiving special education services or special-education -service-related outcomes. It is recommended that future studies further explore the phenomenon observed in this study within other populations.

Another potential limitation of this study was the questionnaire method of data collection utilized in the NHANES program. The exposures and outcomes examined in this study were based on detailed survey questions asked of persons participating in the NHANES program. It is possible that the participants may have recalled information erroneously (e.g., receipt of special education services or receipt of hepatitis B vaccines) or reported information inaccurately (e.g., household income). Despite such limitations, it is presumed that such limitations or errors in the data would have applied equally to all the persons examined. If anything, such errors in the data would have in all probability reduced the overall statistical power of this study to detect true significant relationships.

An additional potential limitation of this study was the method used to determine exposure status within the NHANES program. The exposed populations each were composed of those persons with a record of receiving three doses of hepatitis B vaccine. As previously described, this would seem to be a reasonable assumption because the ACIP has recommended since 1991 that all American children receive the hepatitis B vaccine series as infants [2]. Despite the ACIP recommendation, it is possible that some of the persons assumed to have received three doses of hepatitis B vaccine as infants may have received doses later in life. It is presumed that any doses of hepatitis B vaccine administered later in life would have less or no effect on the risk of a boy receiving special education services. In addition, it was a priori determined that boys who received three doses of hepatitis B vaccine and who were born from 1994–2000 received Thimerosal-containing hepatitis B vaccines, whereas boys who received three doses of hepatitis B vaccine and who were born from 2001–2007 received Thimerosal-reduced vaccines, based upon the previously reported composition of hepatitis B vaccines [6]. It is possible that a small percentage of hepatitis B vaccine doses administered to boys who were born from 1994–2000 may have had no Thimerosal. It is also possible that a small percentage of hepatitis B vaccine doses administered to boys who were born from 2001–2007 were administered Thimerosal-containing hepatitis B vaccines, because existing stocks of Thimerosal-containing hepatitis B vaccines continued to be administered following the Thimerosal phase-out announcement in 1999. It is believed these potential limitations would not have increased the association observed between exposure to Thimerosal-containing hepatitis B vaccines and special education services or decreased the lack of association that was observed between exposure to Thimerosal-reduced hepatitis B vaccines and special education services. In actuality, it is believed that such phenomena would have biased the results observed in the opposite direction (i.e., increased misclassification of exposure status of the populations examined).

A further potential limitation of this study was that it was not possible to know whether the Thimerosal component of the hepatitis B vaccines worked alone or synergistically with other vaccine components to produce the effects observed. In other words, the data support the hypothesis that Thimerosal was necessary for the effects observed, but do not unequivocally establish it as the sole cause. It is presumed from the epidemiological studies previously discussed [10,18,32,33,34] and biological plausibility data that the Hg component of the Thimerosal-containing hepatitis B vaccines was responsible, but it is possible that other components of hepatitis B vaccines, such as the aluminum adjuvant or the antigen, might act synergistically. Future studies should continue to determine which vaccine components may be responsible for special education service outcomes.

Yet a further potential limitation of this study was that there may be differences other than hepatitis B vaccine exposure status when comparing populations receiving three doses of hepatitis B vaccine in comparison to the populations receiving no doses of hepatitis B vaccine. This potential limitation was addressed, in part, by examining the exposed and unexposed populations for contemporaneous time periods. This would tend to minimize factors related to differences in diagnosis and availability of special education services in different time periods. In addition, covariates, such as socioeconomic status and race were also considered, but failed to mediate the associations that were observed in this study. Finally, survey logistic regression models (using stratum, cluster, and weight) were developed to examine the potential relationship between three doses of infant Thimerosal-containing hepatitis B vaccine exposure and the risk of having a stomach or intestinal illness within the last 30 days (variable = HSQ510). The outcome of stomach or intestinal illness within the last 30 days was chosen because it was hypothesized that this condition should not be biologically plausibly linked to Thimerosal exposure from three doses of infant Thimerosal-containing hepatitis B vaccine, and it was presumed to be a measure of the general health status of the populations examined. As revealed in Table 4, exposure to three doses of infant Thimerosal-containing hepatitis B vaccine in comparison to no doses did not increase the risk of stomach or intestinal illness within the last 30 days. This further supports the assertion that there is not systematic bias or confounders mediating the associations observed in this study.

An additional potential limitation of this study was that receipt of special education services was used as the outcome measurement. It was not possible in NHANES to determine the exact type of disability each person was suffering from. This is a potential limitation because a person may receive special education services for a potential wide range of disabilities. It is possible that some disabilities for which a child may receive special education services, such as autism, emotional disturbances, learning disability, speech or language impairment, etc. have a biologically plausible relationship to be linked to increased Hg exposure from Thimerosal-containing hepatitis B vaccines, whereas other disabilities, such as deafness, blindness, traumatic brain injury, orthopedic impairment, etc. do not have a biologically plausible relationship to be linked to increased Hg exposure from Thimerosal-containing hepatitis B vaccines. As a consequence, the observed associations in the present study may under-reflect the extent of the true relationship between Hg exposure from Thimerosal-containing hepatitis B vaccines and specific types of disabilities. It is recommended that future studies attempt to further examine special education services to precisely determine, which types of disabilities are associated or not with increased Hg exposure from Thimerosal-containing hepatitis B vaccine. It is also possible the ancillary factors may affect receipt of special education services, such as changes over years in special education criteria, changes in funding for this educational program, or dominance of neurodiversity ideology—implying normalcy of developmental and learning disabilities. This is why the present study utilized contemporaneous exposed and unexposed groups to minimize the potential impacts of these phenomena on the results that were observed. Future studies should further examine other databases using similar methods for consistency with the results observed in this study.

A final potential limitation of this study was that potential interactions between exposure to Thimerosal-containing hepatitis B vaccine and covariates, such as birth weight, race, socioeconomic status, etc. were not explored. It was observed that the covariates of race and socioeconomic status were significantly related to the receipt of special education services. It is recommended in future studies that potential interactions between Thimerosal-containing hepatitis B vaccine and receipt of special education services be explored.

## 6. Conclusions

This cross-sectional study provides new evidence consistent with and extends the results from previous epidemiological and biological studies on the adverse effects of Hg exposure from Thimerosal-containing childhood vaccines. This study supports a significant about nine-fold increase in the risk of adverse effects as measured by receipt of special education services among boys receiving infant Thimerosal-containing hepatitis B vaccination. Despite the limitation of this cross-sectional study not being able to ascribe a direct cause-and-effect relationship between exposure and outcome, it was estimated that for boys who were born from 1994–2000, exposure to three doses of infant Thimerosal-containing hepatitis B vaccine was associated with an additional approximately 1.2 million boys receiving special education services, and excess education costs of about US $180 billion. By contrast, exposure to Thimerosal-reduced hepatitis B vaccine was not associated an increased risk of receipt of special education services among boys. 

In light of the findings in this study, it is recommended that Thimerosal be removed from all of the vaccines given to pregnant women and children worldwide. This includes the Thimerosal-containing hepatitis B vaccine and all other Thimerosal-containing childhood vaccines that are still being used in the developing world, as well as the Thimerosal-containing influenza vaccines, the Thimerosal-containing meningococcal vaccines, and the Thimerosal-containing tetanus toxoid vaccines that are still used in the US to date. The removal of Thimerosal should take place as soon as possible, since the use of Thimerosal in vaccines is avoidable and unnecessary. Despite the adverse effects that are observed in this study regarding Thimerosal in vaccines, routine childhood vaccination remains an important public health tool to reduce infectious disease morbidity and mortality [35].

## Figures and Tables

**Table 1:** Characteristics of the boys born between 1994 and 2007 (weighted number of boys = 24,537,123) examined in this study.

Parameter Examined	Hepatitis B Vaccine Exposed Group (Weighted Number of Boys = 22,994,525)	Unexposed Group (Weighted Number of Boys = 1,542,598)
Age/Birth Year		
Mean Age ± std (range = 7–8 years old)	7.50 ± 0.50	7.53 ± 0.50
Mean Birth Year ± std (range = 1994–2007)	2000 ± 4.08	2000 ± 4.16
Race (%)		
Non-Hispanic White	14,389,647 (62.58%)	941,656 (61.04%)
Non-Hispanic Black	3,587,239 (15.60%)	219,024 (14.20%)
Hispanic	5,017,639 (21.82%)	381,918 (24.76%)
Status (%)		
Receiving Special Education Services	3,064,785 (13.33%)	127,494 (8.26%)
Socioeconomic Status (score range: 0–5)		
Mean PIR Score ± std	2.31 ± 1.60	2.35 ± 1.56

PIR = poverty income ratio, std = standard deviation of the mean.

**Table 2:** A summary of survey logistic regression models ^1^ generated to examine the potential relationship between infant hepatitis B vaccine exposure and the risk of adverse effects as measured by receipt of special education services.

Model	Variable	Odds Ratio	95% Confidence Interval	*p*-Value
I				
	All Hepatitis B Vaccine Exposure (1–2)	1.707	0.680 to 4.286	0.2551
	***Thimerosal-Containing Hepatitis B Vaccine Exposure (1–2)***	***10.143***	***1.373* to *74.950***	***0.0232***
	Thimerosal-Reduced Hepatitis B Vaccine Exposure (1–2)	0.977	0.348 to 2.744	0.9647
II				
	All Hepatitis B Vaccine Exposure (1–2)	1.686	0.664 to 4.285	0.2722
	Race (non-Hispanic Black vs. non-Hispanic White)	0.734	0.460 to 1.172	0.9932
	***Race (Hispanic vs. non-Hispanic White)***	***0.537***	***0.323* to *0.895***	***0.0417***
	***Socioeconomic Status (0–5)***	***0.787***	***0.653* to *0.950***	***0.0125***
	***Thimerosal-Containing Hepatitis B Vaccine Exposure (1–2)***	***9.234***	***1.306* to *65.286***	***0.0259***
	Race (non-Hispanic Black vs. non-Hispanic White)	0.780	0.416 to 1.463	0.2895
	***Race (Hispanic vs. non-Hispanic White)***	***0.314***	***0.112* to *0.875***	***0.0295***
	Socioeconomic Status (0–5)	0.914	0.735 to 1.136	0.4183
	Thimerosal-Reduced Hepatitis B Vaccine Exposure (1–2)	0.952	0.324 to 2.798	0.9286
	Race (non-Hispanic Black vs. non-Hispanic White)	0.720	0.383 to 1.355	0.5430
	Race (Hispanic vs. non-Hispanic White)	0.737	0.392 to 1.385	0.6245
	***Socioeconomic Status (0–5)***	***0.684***	***0.509* to *0.918***	***0.0114***

^1^ Numbers in parentheses are the NHANES variable codes used in the models. The survey logistic models employed used stratum, cluster, and weight. ***Bold-Italicized*** results are statistically significant. Model I = unadjusted, Model II = adjusted for race and socioeconomic status.

**Table 3:** A summary of the prevalence ratio for the risk of receiving special education services following infant hepatitis B vaccine exposure using survey frequency modeling ^1^.

	Exposed Group	Unexposed Group	Outcome Measurements
**Thimerosal-Containing Hepatitis B Vaccine**			
Weighted Number of Boys Receiving Special Education Services (%)	1,444,588 (12.91%)	10,147 (1.44%)	
Weighted Total Number of Boys	11,186,579	704,254	
Prevalence Ratio (95% CI)			***8.961 (1.202 to 66.667)***
*p*-value			***0.006***
Prevalence Attributable Rate			***0.1147***
**Thimerosal-Reduced Hepatitis B Vaccine**			
Weighted Number of Boys Receiving Special Education Services (%)	1,620,196 (13.72%)	117,347 (14.00%)	
Weighted Total Number of Boys	11,807,946	838,344	
Prevalence Ratio (95% CI)			0.980 (0.396 to 2.426)
*p*-value			0.9649
Prevalence Attributable Rate			–0.00276

^1^ The survey frequency model employed used stratum, cluster, and weight. ***Bold-Italicized*** results are statistically significant. This table reveals that an estimated 1,283,101 boys born from 1994–2000 received special education services attributably associated with receipt of three doses of infant Thimerosal-containing hepatitis B vaccine. Assuming such persons received 15 years of special education services; the estimated excess overall education cost was US $180,320,599,035.

**Table 4:** A summary of survey logistic regression models ^1^ generated to examine the potential relationship between infant hepatitis B vaccine exposure and the risk of having a stomach or intestinal illness within the last 30 days.

Model	Variable	Odds Ratio	95% Confidence Interval	*p*-Value
I				
	Thimerosal-Containing Hepatitis B Vaccine Exposure (1–2)	0.816	0.241 to 2.764	0.7434
II				
	Thimerosal-Containing Hepatitis B Vaccine Exposure (1–2)	0.810	0.239 to 2.743	0.7350
	Race (non-Hispanic Black vs. non-Hispanic White)	0.437	0.167 to 1.145	0.0532
	Race (Hispanic vs. non-Hispanic White)	0.985	0.417 to 2.327	0.2919
	***Socioeconomic Status (0–5)***	***0.781***	***0.648 to 0.941***	***0.0092***

^1^ Numbers in parentheses are the NHANES variable codes used in the models. The models employed used stratum, cluster, and weight. ***Bold-Italicized*** results are statistically significant. Model I = unadjusted, Model II = adjusted for race and socioeconomic status.
