# Addendum: Geier, D.A.; et al. A Cross-Sectional Study of the Association between Infant Hepatitis B Vaccine Exposure in Boys and the Risk of Adverse Effects as Measured by Receipt of Special Education Services. *Int. J. Environ. Res. Public Health* 2018, *15*, 123

**DOI:** 10.3390/ijerph15061141

**Published:** 2018-06-01

**Authors:** David A. Geier, Janet K. Kern, Kristin G. Homme, Mark R. Geier

**Affiliations:** 1 Institute of Chronic Illnesses, Inc., Silver Spring, MD 20905, USA; davidallengeier@comcast.net (D.A.G.); mgeier@comcast.net (M.R.G.); 2 CoMeD, Inc., Silver Spring, MD 20905, USA; 3 CONEM US Autism Research Group, Allen, TX 75013, USA; 4 International Academy of Oral Medicine and Toxicology, Champions Gate, FL 33896, USA; khomme@sbcglobal.net

The authors would like to update the “Conflicts of Interest” section of their previous paper [1] as follows:
